# Meat Quality Assessment by Electronic Nose (Machine Olfaction Technology)

**DOI:** 10.3390/s90806058

**Published:** 2009-07-30

**Authors:** Mahdi Ghasemi-Varnamkhasti, Seyed Saeid Mohtasebi, Maryam Siadat, Sundar Balasubramanian

**Affiliations:** 1 Agricultural Machinery Engineering Department, University College of Agriculture and Natural Resources, University of Tehran, P.O. Box 4111, Karaj 31587-77871, Iran; E-Mail: mohtaseb@ut.ac.ir; 2 Laboratoire Interfaces Composants et Microélectronique, LICM/CLOES/SUPELEC, Université de METZ 2, Rue E. Belin, 57070 METZ, France; E-Mail: siadat@univ-metz.fr; 3 Department of Biological and Agricultural Engineering, Louisiana State University, AgCenter, 149 E.B. Doran Building, Baton Rouge, LA 70803, USA; E-Mail: sbalasubramanian@agcenter.lsu.edu

**Keywords:** meat, spoilage, electronic nose, sensor, quality control, machine olfaction, neural network

## Abstract

Over the last twenty years, newly developed chemical sensor systems (so called “electronic noses”) have made odor analyses possible. These systems involve various types of electronic chemical gas sensors with partial specificity, as well as suitable statistical methods enabling the recognition of complex odors. As commercial instruments have become available, a substantial increase in research into the application of electronic noses in the evaluation of volatile compounds in food, cosmetic and other items of everyday life is observed. At present, the commercial gas sensor technologies comprise metal oxide semiconductors, metal oxide semiconductor field effect transistors, organic conducting polymers, and piezoelectric crystal sensors. Further sensors based on fibreoptic, electrochemical and bi-metal principles are still in the developmental stage. Statistical analysis techniques range from simple graphical evaluation to multivariate analysis such as artificial neural network and radial basis function. The introduction of electronic noses into the area of food is envisaged for quality control, process monitoring, freshness evaluation, shelf-life investigation and authenticity assessment. Considerable work has already been carried out on meat, grains, coffee, mushrooms, cheese, sugar, fish, beer and other beverages, as well as on the odor quality evaluation of food packaging material. This paper describes the applications of these systems for meat quality assessment, where fast detection methods are essential for appropriate product management. The results suggest the possibility of using this new technology in meat handling.

## 1. Introduction

The main sensory system used by humans to sense flavor is olfaction; therefore if the flavor of a particular substance is to be characterized, the use of smell can often provide us with suitable information [1]. In order to understand the operation of an “electronic nose” we must first analyse what is involved in “smelling” and therefore what constitutes a “smell”, i.e., an odor. Odorant molecules have some basic characteristics, the primary ones being that they are light (relative molecular masses up to approximately 300 Da), small and polar and that they are often hydrophobic. A simple odor, for example an alcohol, contains only one chemical component. A complex odor is a mixture of many different odorant molecules each in varying concentration; for example, the headspace of coffee is made up of hundreds or even thousands of different molecules.

Significant progress has been made in the objective electronic registration and processing of information which is gathered subjectively by the human eye, ear, or touch senses. However, the present state of the art to record or to mimic electronically human olfaction and taste senses is characterized by completely inadequate and very preliminary approaches [2,3]. Based on Ziegler *et al.* [4], there are a variety of reasons for this. As one example, all attempts to compose complex odors by well defined amounts of a limited number of standard primary odors failed so far. Other reasons are the lack of an ‘odorant vector space’ and of a direct correlation between chemical structure and odor perception. Genetic differences of an estimated 1,000 olfactory receptor genes in humans influence the individual variations in odor perception. Furthermore, every person has unique experience helping to determine how to react to specific chemosensory events.

**Figure 1.**
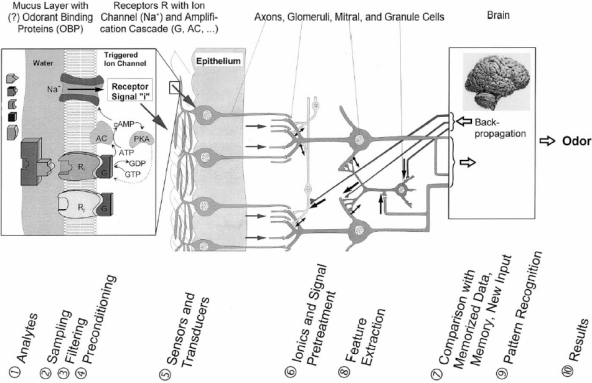
Human nose: schematic representation of ten different components in the signal cascade of the human nose to recognize odor molecules (indicated as “analytes”). The input from odorant binding proteins on the overall molecular recognition process is an active area of current research. As shown on the below of this figure, different stages is followed to determine an analyte, e.g., in number 2, sampling in human olfactory system is done by nose.

The general sensation of odors in biology not only includes human odor sensation but also the odor sensation of animals in air and in water with their completely different sensitivity and selectivity patterns to detect chemical species in the gas and liquid state. Since we do not know details of odor perception in the various animals, the most general definition of a nose is a detection system to sense any molecule in the gas or liquid state. The odor patterns generated by such odorant detector systems evidently depend on their biological and biochemical architecture (see schematic Figure 1). These also depend on the individual training, learning, and preconditioning of the systems, on the environment, etc.

The perception of volatile compounds by the human nose is of great importance in evaluating the quality of foods, cosmetics and numerous other items of everyday life. Therefore, it is not surprising that repeated efforts have been made over the years to introduce instruments operating on a similar principle as the human nose. These systems would in most cases not replace but complete conventional analyses of volatile compounds by sensory methods and by traditional analytical techniques.

## 2. The Concept of Electronic Nose (E-nose)

The term Electronic Nose is understood to describe an array of chemical gas sensors with a broad and partly overlapping selectivity for measurement of volatile compounds within the headspace over a sample combined with computerised multivariate statistical data processing tools [5]. The electronic nose has derived its name because it in several aspects tries to resemble the human nose. Human olfactory perception is based on chemical interaction between volatile odor compounds and the olfactory receptors (primary neurons) in the nasal cavity. The signals generated are transferred to the brain through synapses and secondary neurons and further led to the limbic system in the cortex where identification of odor takes place based on neural network pattern recognition. In principle, the primary neurons correspond to the chemical sensors of the electronic nose with different sensitivity to different odors. By chemical interaction between odor compounds and the gas sensors the chemical state of the sensors is altered giving rise to electrical signals which are registered by the instrument analogue with the secondary neurones. In this way the signals from the individual sensors represent a pattern which is unique for the gas mixture measured and is interpreted by multivariate pattern recognition techniques like artificial neural network, the brain of the instrument. Samples with similar odors generally give rise to similar sensor response patterns and samples with different odors show differences in their patterns. When the sensor patterns for a series samples are compared, differences can be correlated with the perceived sample odor.

As demonstrated in a paper by Haugen and Kvaal [6], the sensor array of an electronic nose has a very large information potential and will give a unique overall pattern of the volatiles. In principle, both the electronic and the human nose operate by sensing simultaneously a high number of components giving rise to a specific response pattern. However, there are two basic differences between the human and the electronic nose that should be kept in mind. The electronic nose has both large differences in sensitivity and selectivity from the human nose. The sensors of an electronic nose respond to both odorous and odorless volatile compounds. Taking these constraints into consideration in the choice of sensors used for these instruments it is possible to design an electronic nose with a response similar to the human nose for specific compounds. Still, the mechanisms involved will be fundamentally different. In principle, the electronic nose can be applied to any product that gives off volatiles with or without smell provided that this occurs within the sensitivity range of the sensors. As sensor technology plays a crucial role in reaching the desired analytical qualities, the following review concentrates on discussing the present status of sensor technology and statistical data analysis. Particular reference is then made to applications of electronic nose systems to meat.

## 3. Sensor Array Technology

An electronic nose comprises a sensor array using several “chemosensors” and a computer. The different types of chemosensors, especially odor sensors which have been employed within an e-nose are described in this section.

A chemosensor is a device that is capable of converting a chemical quantity into an electrical signal and thus respond to the concentration of specific particles such as atoms, molecules, or ions in gases or liquids by providing an electrical signal. Chemosensors are very different from physical sensors. Although approximately 100 physical measurands can be detected using physical sensors, in the case of chemosensors this number is higher by several orders of magnitude. The types of chemosensors that can be used in an e-nose need to respond to odorous molecules in the gas phase, which are typically volatile organic molecules with different relative molar masses [3].

The ideal sensors to be integrated in an electronic nose should fulfill the following criteria [7]: high sensitivity towards chemical compounds, that is, similar to that of the human nose (down to 10^−12^ g/mL); low sensitivity towards humidity and temperature; medium selectivity, they must respond to different compounds present in the headspace of the sample; high stability; high reproducibility and reliability; short reaction and recovery time; robust and durable; easy calibration; easily processable data output; small dimensions.

**Figure 2.**
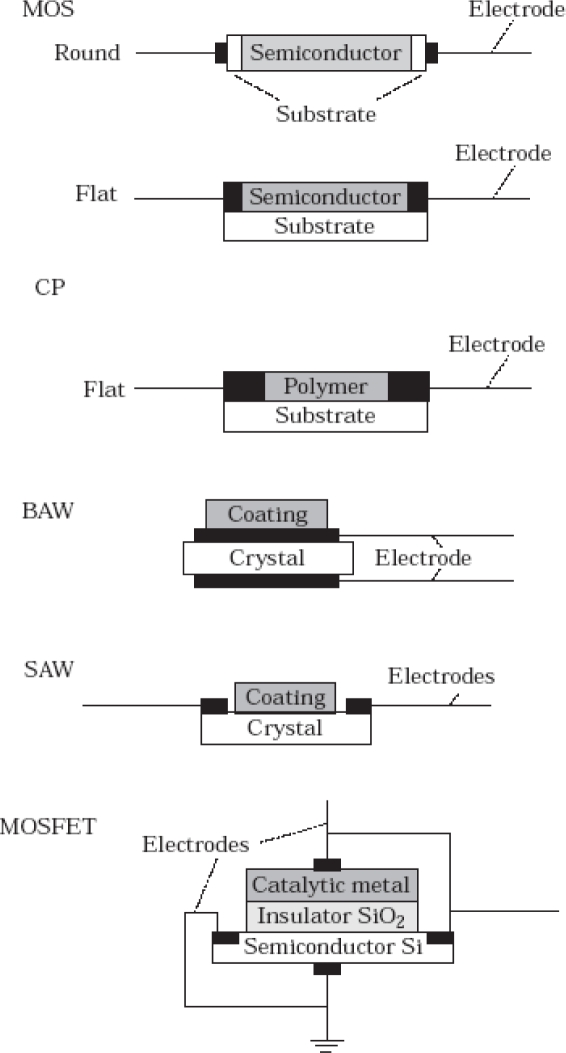
Schematic diagrams of 5 different kinds of sensors. Caption: MOS = Metal oxide semiconductor; CP = Conducting polymer; BAW = Bulk acoustic wave; SAW = Surface acoustic wave; MOSFET = Metal oxide semiconductor field effect transistor.

As the sensors are designed for industrial purposes, especially as on-line systems, a minimal working temperature with low power consumption, a high safety level, and low manufacturing costs present valuable advantages. Most manufacturers are looking for highly selective sensors. In the case of an electronic nose, every compound present in the gaseous phase should be detected by at least one sensor. If a new compound is added to a mixture, at least one sensor must detect this addition. The use of too many sensors leads to an over complex system with a large amount of unnecessary data. Various kinds of gas sensors are available, but only four technologies are currently used in commercialized electronic noses (Figure 2): metal oxide semiconductors (MOS); metal oxide semiconductor field effect transistors (MOSFET); conducting organic polymers (CP); piezoelectric crystals (bulk acoustic wave = BAW).

## 4. Analysis Techniques

As can be deduced, a huge amount of data is generated at the input level of biological as well as biohybrid or artificial sensor systems. This requires complex data evaluation methods which will be characterized briefly in the following.

In electronic noses pattern recognition methods are required for the qualitative analysis of odors or of different compounds present in a certain mixture and multicomponent analysis methods are required for the quantitative determination of one or more compounds in a mixture. In all chemometric approaches experimental data are evaluated by a qualitative or quantitative link between output signals of an instrument and the chemical information (composition or concentration of analytes). This requires a comparison of the sensor outputs with previously recorded calibration data. These calibration data consist of measurements in which concentrations of pure analytes or mixtures of analytes are correlated with the corresponding sensor signals. This correlation is described by a calibration function which should make it possible to analyze unknown samples (containing only components included in the calibration) with sufficient accuracy (with small systematic errors) and reproducibility (with small standard deviations) concerning corresponding predictions.

**Figure 3.**
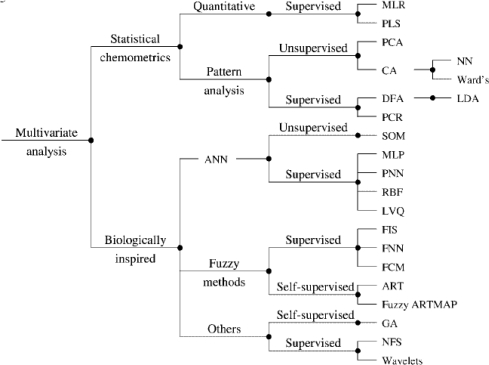
Classification scheme of the multivariate pattern analysis techniques applied to EN data. MLR (Multiple linear regression), PLS (Partial Least Square), PCA (Principles Component Analysis), CA (Cluster Analysis), DFA (Discrimination Functional Analysis), PCR (Principles Component Regression), SOM (Self Organizing Maps), MLP (Multilayer Perceptron), RBF (Radial Basis Function),… for more abbreviations, the reader is referred to Pearce *et al.* [3].

Multivariate data analysis, as demonstrated in Figure 3 generally involves data reduction, it reduces high dimensionality in a multivariate problem where variables are partly correlated (e.g., sensors with overlapping sensitivities), allowing the information to be displayed in a smaller dimension (typically two or three). Some information on the items and abbreviations shown are presented as follows.

**Figure 4.**
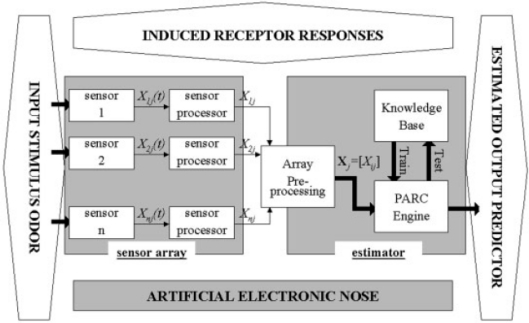
Basic architecture of a data processing system for an EN.

There are many multivariate techniques to choose from. A subset called pattern recognition (PARC) techniques is routinely used in electronic nose data analysis. The responses generated by an array of odor sensors may be processed using a variety of techniques. In Figure 4, where the basic data-processing structure of an EN is presented, the array formed from the sensor outputs is pre-processed and normalized so that the modified response matrix can be fed into a PARC engine.

The nature of a PARC engine is usually classified in terms of being parametric or nonparametric, and supervised or unsupervised.

*Parametric*: A parametric technique, commonly referred to as a statistical approach, is based on the assumption that the spread of the sensor data can be described by a probability density function (PDF). In most cases, the assumption made is that the data follow a normal distribution with a constant mean and variance. These techniques try to find an underlying mathematically formulated relationship between system inputs, odor vectors and its outputs, classes or descriptors.

*Non-parametric*: Non-parametric methods do not assume any specific PDF for the sensor data and thus apply more generally. This approach to multivariate data analysis has led to the fields of artificial neural networks (ANNS) and expert systems.

*Supervised:* In a supervised learning PARC method, a set of known odors are systematically introduced to the EN, which then classifies them according to known descriptors or classes held in a knowledge base. Then, in a second stage for identification, an unknown odor is tested against the knowledge base, now containing the learnt relationship, and then the class membership is predicted. Unknown odor vectors are analyzed using relationships found a priori from a set of known odor vectors used in an initial calibration, learning, or training stage. The idea of testing a method using unclassified response vectors is well established and is often referred to as cross-validation.

*Unsupervised:* For unsupervised learning, PARC methods learn to separate the different classes from the response vectors routinely, discriminating between unknown odor vectors without being presented with the corresponding descriptors. These methods are closer to the way that the human olfactory system works using intuitive associations with no, or little, prior knowledge.

*Linear/non-linear*: The above multivariate analyses are all linear PARC methods where a model is calculated using linear combinations of input data [8]. Most sensors have a non-linear response versus concentration; however, these techniques work well if a low concentration of volatiles ensures an approximately linear response. In addition, the use of pre-processing algorithms, such as averaging, linearization or normalization, improves the performance of these analytical techniques [9]. When high concentrations of volatile are measured, a non-linear PARC technique, such as ANN or RBF, would be more appropriate. Non-linear models usually need more parameters, since some of them are used to describe the shape of the non-linearity (more input data than linear models). The main advantage of such a method is flexibility, i.e. the ability to adjust to more complex data variations. However, caution is necessary when choosing model flexibility; this can be achieved by selecting the number of parameters. If too many parameters are taken into account, the calculated model will be over-flexible, fitting to all relevant data variations and unwanted sensor noise. The best method to avoid an over-fitted model is to use training data to build a non-linear model, and validation data to test this model (cross-validation).

### 4.1. Artificial Neural Network

**Figure 5.**
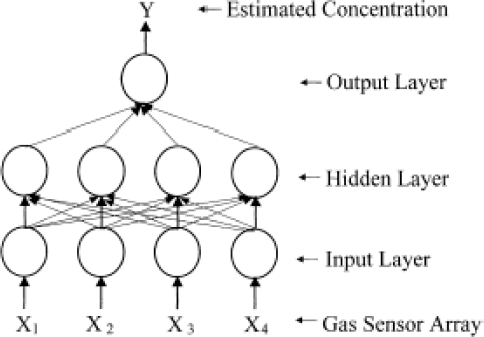
ANN architecture of e-nose. The first layer is called as gas sensor layer; the second, hidden layer; the last, output concentration layer. Signals are transferred from layer to layer and their values are decided by both the transfer function used by each neuron and the connection weights between two different neurons.

As shown in Figure 5, a neural network consists of a set of interconnected processing algorithms functioning in parallel. On a very simplified and abstract level, ANN is based on the cognitive process of the human brain. Mathematical functions, or neurons, link together to build a network which mimics the human nervous system [10].

A weight is randomly assigned to each neuron and then adjusted by means of an iterative or “learning” process, for example, error back-propagation, until the desired outputs are obtained. The resultant set of weights and functions is then saved as a “neural network”. Like CDA, ANN is a supervised method and so needs a minimum of known data to correctly train the system. If the number of available data is not sufficient an erratic result will be obtained [11]. Unlike other PARC methods, a neural network is a dynamic, self-adapting system that can modify its response to external forces using previous experience, offering a more flexible and, due to the parallelism, faster method of analysis. In addition, it may more closely mimic mammalian neuron processing of odor stimuli. A well trained ANN is very efficient in comparing unknown samples to a number of known references.

Referring to [4], the reason ANNs have become so attractive in a number of scientific disciplines seems to be twofold: First, they are an attractive approach to modeling parts of the biological nervous system, although ongoing research shows that this modeling is much too simplistic. But more important, ANNs are a powerful and relatively simple method for data driven pattern analysis, high dimensional function interpolation and time series prediction, which by different network design and different raining algorithms may be adapted for a large number of tasks.

The main advantages of ANNs are:

*Trainability*: ANNs may be trained from training patterns (examples) by automated learning procedures to fulfill their task, thus they can easily be adapted to new data. *Parallelism*: ANNs are inherently parallel and may be implemented on parallel hardware, from workstation clusters over MIMD and SIMD computers up to neurocomputers.
*Simplicity*: The network structure and learning algorithms of the most popular ANNs may easily be described and understood. Many rival methods require a deeper knowledge of the underlying mathematics to apply them properly.
*Noise tolerance*: if trained to this aim (but only then!), ANNs may show a large degree of robustness against noise or corrupted input data.
*Automatic generalization*: ANNs may extract the typical characteristics of the input data, thus supplying missing features with default values and offering automatic generalization to common features.
*High performance*: for pattern recognition tasks, ANNs rival and often surpass the best known conventional (e.g., statistical) techniques, especially for problems with a large number of training data.

However, ANNs also possess a number of inherent disadvantages:

*Dependence on learning*: NN knowledge may not easily be augmented or exchanged, except by learning algorithms. There is no exchangeable or extendible knowledge base as in expert systems.
*Black box characteristics*: Despite the theoretical analyses in the last years, which yielded capability results for networks classes, a trained neural network of moderate size is still not as easily analyzed as other representations (rules, frames, logic, and fuzzy rules).
*Problems with sequential reasoning*: Logical reasoning can be done in principle with ANNs, but here other, symbolic AI approaches, are much better suited.
*Time-consuming learning*: Many NN learning algorithms are very time-consuming, especially if the network size and the number of training patterns is large.

### 4.2. Graphical Methods for Exploratory Analysis

Sensor arrays may generate a large volume of high dimensional data; it is often a challenge to extract useful information from the data to solve the problem under investigation. Graphical methods are a simple exploratory way of analysing data, some methods may plot the high-dimensions produced, whilst others reduce the data to two or three dimensions for visual analysis.

*Bar charts:* These are a simple and useful way of visualizing the sensor response patterns that are produced by a sensor array. The sensor responses are plotted as a height value with a small width, next to each other.

Individual or the mean of several samples are plotted for each analyte. An estimation of the effectiveness and composition of the array may then be made based on the patterns produced. Walmsley *et al.* [12] used bar charts of the response of 4 MOS sensor array to show that the patterns produced were different for each of 6 analytes. McAlernon *et al.* [13] used 3D bar charts, PCA and multivariate analysis of variance (MANOVA) to interpret the frequency change patterns of an array of 8 TSM sensors for o-xylene, toluene, dodecane and tetradecane. Park and Zellers [14] used bar charts to show the relative response patterns of polymer coated SAW sensors to toluene and trichloroethylene. Groves and Zellers [15] analysed solvent vapours in breath and ambient air with an array of four polymer coated SAW sensors. Time response curves and bar charts were used to show the relative response patterns for 16 solvents. The bar charts provided a visual indication of the discriminating capability of the array to the various solvents.

**Figure 6.**
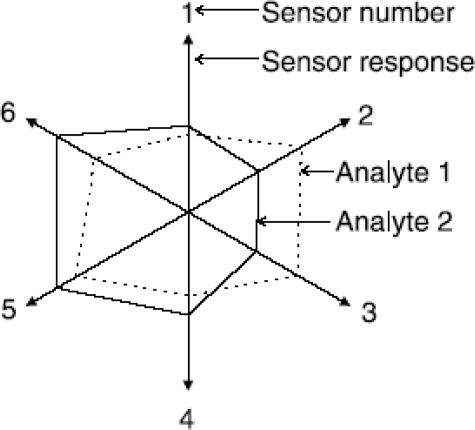
A polar plot of sensor response data.

*Polar plots:* Polar plots or radar plots a.k.a Star plots [16] display multivariate dimensional data in two dimensions for exploratory analysis. Axes for each feature radiate from the origin at equal angles with the magnitudes of the features joined by straight lines. Figure 6 shows a typical plot for a 6 sensor array. Some pre-processing of the data may exaggerate small difference in shapes so that they may be more easily seen.

For example a reference data set may be subtracted from the feature vectors and rescaled to emphasis the differences. Brezmes *et al.* [17] used polar plots to compare both the difference between repeated measurements and measurements of different aromatic species; cinnamon, red pepper, thyme, pepper and nutmeg. The method showed large differences in the shapes for different species, and possible poor reproducibility by smaller differences between repeated runs of the same groups. Jonsdottir *et al.* [18] characterised the flavors of ripened cod roe by polar plots utilising the results of a sensory panel. From this an array of 4 EC sensors were used to examine the same cod roe samples. Gan *et al.* [19] characterised responses to palm olein and vegetable oils from a virtual array of SAW sensors, based on a single sensor responding to the analyte being desorbed from a GC column trap, plotting the raw frequency shift as amplitude and the time as the angle. PCA scores were plotted to demonstrate the separation achieved but no classification algorithm was tried.

**Figure 7.**
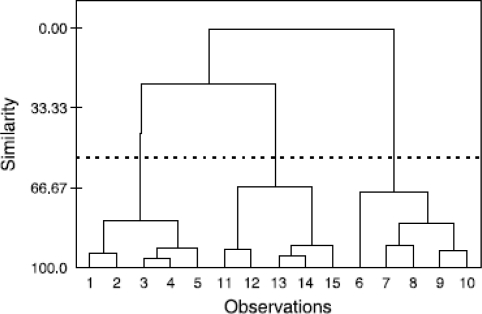
Dendogram illustrating HCA data clustering.

*Hierarchical cluster analysis (HCA)*: These techniques attempt to separate data into specific groups [20], based on a similarity measure. Initially each data point represents its own cluster, then the threshold for the decision when to declare two or more objects to be members of the same cluster is lowered incrementally. As a result more and more objects are linked together and aggregated into larger and larger clusters of increasingly dissimilar elements. Finally, all objects are joined together. The result of hierarchical clustering methods may be displayed as a dendrogram, as shown in Figure 7; the vertical axis denotes the similarity. With classification set at a similarity level of 50%, the data has been classified into 3 groups. There are many types of similarity linkage, the three most common are single linkage (nearest neighbour), the distance between two clusters is determined by the distance of the two closest objects in the clusters.

Complete linkage (furthest neighbor), the distances between clusters is determined by the greatest distance between any two objects in the different clusters. Group average, the distances between clusters is determined by the mean distance of objects in different clusters.

*Sammon mapping*: This is a nonlinear mapping (NLM) technique [21] that attempts to preserve the structure of the data in the projected space from the original high dimensional space by maintaining the distances between points under projection [22]. The distance metric usually employed is the Euclidean, although others have been used [23]. If the distance between two points i and j in the input space of vector x is d^*^_ij_ = *d*(*x_i_*,*x_j_*) and the distance between the points in projected space of vector y is *d_ij_* = *d*(*y_i_*,*y_j_*) Sammon suggested looking for values of y to minimize an error function called the mapping stress E:

$$E=\frac{1}{\sum_{i=1}^{n-1}\sum_{j=i+1}^{n}d_{\mathrm{ij}}}\sum_{i=1}^{n-1}\sum_{j=i+1}^{n}\frac{{\left(d_{\mathrm{ij}}^{*}-d_{\mathrm{ij}}\right)}^{2}}{d_{\mathrm{ij}}^{*}}$$

Sammon used a method of steepest descent for (approximate) minimisation of E but there are many local minima on the error surface and it is unavoidable for the algorithm to become stuck. The algorithm is usually run several times with different initial configurations and the outcome with the lowest stress chosen. There is a high computational load, which is O(n^2^). At every iteration n(n−1)/2 distances and error derivatives must be calculated. As n the number of patterns increases the computational requirements increase quadratically.

## 5. Applications to Meat

Meat, especially beef, reaches an acceptable state for consumption after a long period of storage at low temperature, a storage procedure known as aging. During storage, not only aging but also bacterial spoilage can occur. Consequently, to obtain appropriately aged meat, it is desirable to monitor the progress of aging and bacterial spoilage simultaneously. For this purpose, a work entitled “direct evaluation of meat spoilage and the progress of aging using biosensors” was planned by Yano *et al.* [24]. They developed a direct sensing method for monitoring meat quality. The sensor is composed of an Ag/AgCl electrode and a platinum electrode on which putrescine oxidase or xanthine oxidase were immobilized to estimate bacterial spoilage or the progress of aging, respectively. A potential-step chronoamperometric method was applied in which the potential was stepped from 300 mV to 600 mV. A linear relationship was obtained between 5 and 60 nmol g^−1^ for putrescine (Put) and 0.05 and 1.0 μmol g^−1^ for hypoxanthine (Hx). The coefficient of variation was 0.75% for 20 nmol mL^−1^ Put solution and 2.2 for a meat sample using the putrescine sensor, and 1.09% for 0.25 μmol mL^−1^ Hx solution and 2.6% for a meat sample using the xanthine sensor. The pH requirements and substrate selectivity were suitable for the direct measurement of substrates on the surface of meat. From the results of practical experiments, the direct sensing method was indicated to be useful with some modifications for the estimation of meat quality during aging. This method is capable of comparison with the electronic nose method (gas-sensor array). In principle, the results obtained from a gas-sensor array represent qualitative and quantitative information of the composition of the headspace gas mixture of a sample. The technique should therefore have a great potential in a number of applications related to meat. Quality control is of great importance within the meat industry and with this technique it would be possible to monitor the meat from the raw material throughout the process and to the final product by analysing volatile compounds released from the meat matrix. There are several aspects of quality control that may be the issue in the context of meat; Sensory quality, shelf life, spoilage, off-flavor and taints and authenticity. In addition, the electronic nose may have a potential in product development when it comes to the design of a product with certain flavor characteristics.

Within food, the majority of publications refer to meat products. Most of the studies were carried out by Berdague at the Station de Recherches sur la Viande at INRA Theix, Clermont-Ferrand (France). Berdague’s studies used instruments based on MOS sensors, starting in 1993 with the Alabaster-UV, one of the first commercially available instruments. This system consists of a stainless steel measurement chamber containing one semiconductor gas sensor, a UV-lamp, and air inputs and outputs connected to a fan. The signal obtained is shown as desorption curves in “Alabaster” units. The first study was carried out in collaboration with Talou of the Ecole Nationale Superieure de Chimie de Toulouse (France). It was shown that this simple instrument could differentiate maxima in odor perception resulting from the maturation of dry nonspiced sausages and rapidly detect sex-linked differences in meat product composition [25]. This research group also initiated a study into the discrimination of micro-organisms using a multi-sensor MOS instrument [26]. Analysing a headspace generated by bacteria grown on agar medium, the FOX 2,000 correctly classified 90.5% of the analysed bacteria species [27]. Bacterial strains commonly used for curing, or pathogenic strains sometimes found in meat products were also detected and correctly classified. Six types of French dry-sausages and 16 Iberian hams, both with and without off-flavor were also correctly identified [28]. Another study, conducted over three days, demonstrated that non-controlled ambient air, which simulated an on-line quality control, could be used in the rapid discrimination of food products. Research was carried out on sausages and the data obtained subjected to modeling by the *Gompertz sigmoidal function*, and discriminant analysis with backward variable selection. In both cases satisfactory results were achieved, with 100% and 97% product recognition respectively [29].

Winquist *et al.* [30] also studied the performance of an electronic nose with meat products. Using an NST 3210 Emission Analyser, samples of ground beef and pork that had been stored in a refrigerator between analyses were studied using ANN and an algorithm based on an abductory induction mechanism. Both types of pattern recognition software could predict the type of meat, but storage time was more problematic. Neural networks based on unsupervised training methods could be used for a storage time prediction of ground beef. Conversely, Shiers *et al.* [31] did not succeed in monitoring the spoilage of beef mince using a CP system. This failure was explained by a high sensitivity of CP sensors to moisture and a low sensitivity to the small quantity of volatile species generated during meat spoilage.

Braggins and Frost [32] looked at the odor and flavor of raw and cooked minced meat of lamb of extended chilled storage in CO_2_ atmosphere and frozen vacuum packed storage by using a sensory panel and a commercial electronic nose (Alpha MOS Fox 4000). The sensor array consisted of 18 different MOS sensors. By using canonical discriminant analysis they could reliably distinguish between lamb mince samples of different storage conditions over a period from 4 to 14 weeks. Eklov *et al.* [33] made a comparison between sensory analysis and electronic nose of fermented sausages. The gas sensor array used consisted of 10 MOSFET and four Taguchi (MOS) gas-sensors. Both techniques were sensitive to small quality differences between batches of sausages and there was a good agreement in discrimination capability between the two techniques. These examples demonstrate that the electronic nose may have the ability to describe and predict sensory properties of meat characteristics. However, it should be emphasized that the result from the sensory analysis always will be the ultimate answer when it comes to characterize flavor of a food product. Provided that the instrument has been calibrated against sensory analysis and performs according to the required sensory properties, accuracy and reproducibility, it could be applied to partly replace a sensory panel in the industry [6].

Electronic noses have been widely used to distinguish between “spoiled” and “unspoiled” meat products. Spoilage of meat can be considered as an ecological phenomenon that encompasses the changes of the available substrata (e.g., low molecular compounds) during the proliferation of bacteria that consists the microbial association of the stored meat. The prevailing of a particular microbial association, of meat depends on factors that persist during processing, transportation and storage in the market. It is well established that in any food ecosystem includes five categories of ecological determinants (e.g., intrinsic, processing, extrinsic, implicit, and the emergent effects). These influence the establishment of the particular microbial association and determine the rate of attainment of a climax population so called (by the) “Ephemeral/specific spoilage micro-organisms -E(S)SO”, i.e., those which are able to adopt various ecological strategies [34,35]. These ecological strategies, developed by the ESO, are the consequence of environmental determinants (e.g., stresses, destructive or enrichment disturbance of the ecosystem, the availability of energy or oxygen competitors), and allow them to proliferate in all available niches. In fact, all of the determinants mentioned above constitute a virtual ecological niche or in other words an n-dimensional hypervolume or hyperspace cloud (HSC) in which an organism influence in (micro) space and time [36]. Indeed the ecosystem approach is pertinent in an analysis of changes occurring in fresh meat during the distribution chain. Therefore, in practice, scientists and technologists involved in meat industries attempt to control (e.g., temperature) or modify some or all of the parameters noted above in order either to extend the shelf life of meat or to create new products with acceptable shelf life [37].

**Figure 8.**
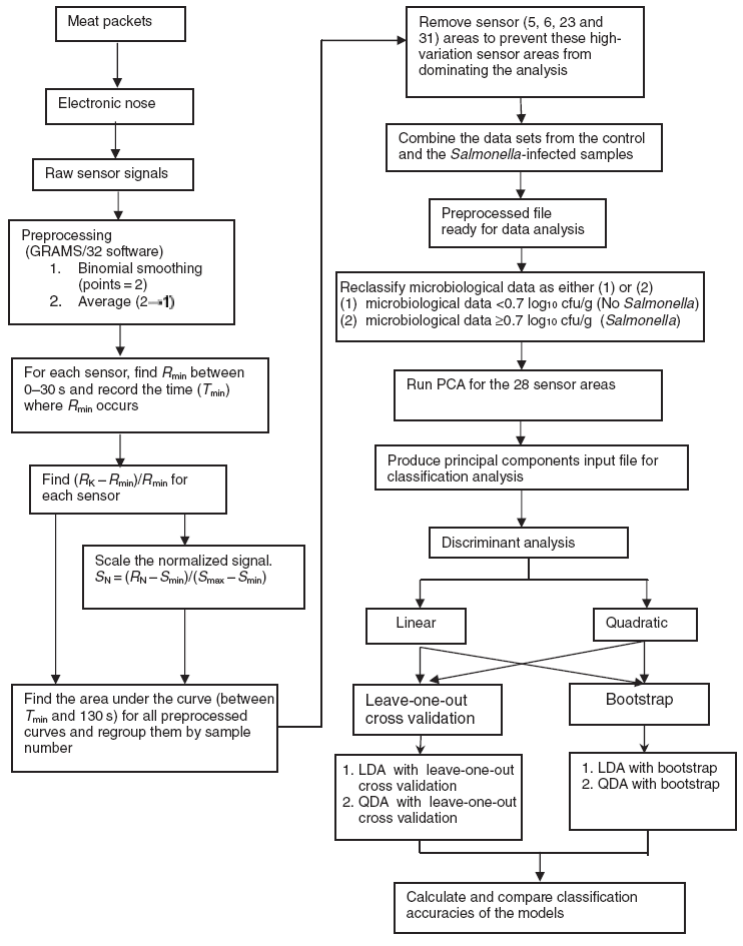
PCA, principal component analysis; LDA, linear discriminant analysis; QDA, quadratic discriminant analysis were used to develop classification models in order to determine beef quality [39].

Electronic nose techniques have also been used on several applications concerned with classification of meat samples. Balasubramanian *et al.* [38] demonstrated the use of linear discriminant analysis (LDA) and quadratic discriminant analysis (QDA) techniques on principal component analysis (PCA)-reduced data sets for discriminating spoiled beef samples from unspoiled ones. They reported the highest classification accuracies of 97.4% and 98.5% for meat samples stored at 10 °C and 4 °C, respectively, when QDA and bootstrapping techniques were used for the classification. The incidence of pathogenic microorganisms, including *Salmonella* in meat products, is a serious threat to consumer safety, and hence, needs to be detected early. For this purpose, a research work was undertaken by Balasubramanian *et al.* [39] to evaluate the performance of a commercially available electronic nose system to discriminate between Salmonella-contaminated beef meat samples and uncontaminated ones. In their work, A commercially available Cyranose-320™ (Cyrano Sciences, Pasadena, CA) conducting polymer–based electronic nose system was used to analyze the volatile organic compounds emanating from vacuum-packaged beef strip loins (*Longissimus lamborum*) that were repackaged simulating retail store conditions and stored at 4 °C and 10 °C after inoculating them with *Salmonella typhimurium*. Two statistical techniques, i.e., linear discriminant analysis (LDA) and quadratic discriminant analysis (QDA), were used to develop classification models from the collected sensor signals (Figure 8).

The results obtained prove that the electronic nose system could identify meat samples contaminated with *Salnonella typhimurium* at a population concentration level of 0.7–2.6 log_10_ cfu/g. Siegmund and Pfannhauser [40] used an electronic nose to study the changes of the volatile fraction of heat-treated chicken meat during storage. They compared the results obtained in this study with those obtained with a GC-MS and GC olfactometry. The smell patterns obtained using the electronic nose was analyzed using cluster analysis. Their results showed high correlation between the results obtained with the electronic nose with that obtained using the GC-MS and GC olfactometry. For their experiments, Siegmund and Pfannhauser [40] used an electronic nose consisting of a sensor array having 32 conducting polymer sensors. The sensor temperature was 35 °C. However, the authors reported that a principal component analysis did not differentiate cooked chicken meat samples, which, were stored for 24–48 hours at 4 °C, and the samples, which were not stored to a good extent. They therefore performed a PCA analysis of the discriminant factors between the different data classes. This procedure minimized the variances between the datasets and maximized the Euclidean distances between the datasets, effecting a better separation. By this mathematical procedure, Siegmund and Pfannhauser [40] achieved better differentiation between samples stored at different times.

Annor-Frempong *et al.*, [41] studied the response of an electronic nose to various intensities of “boar taint”. They validated their results from the electronic nose with that obtained from a sensory panel and a GC. While analyzing their data, they first used a Canonical correlation approach to visualize the relationship between the GC measurements of two indicator odor compounds with those from the e-nose measurements or sensory panel measurements. Later, they employed a multivariate discriminant analysis method to classify the odors based on Fisher’s linear discriminant function. This supervised pattern recognition routine was employed to classify the boar taint based on concentrations and responses. By their approach they obtained a 90% correct classification rate in the training data set for classification based on concentrations and about 53% for the testing data set. However, they obtained 100% correct classification based on responses for the training set data and 84.2% correct classification for the testing set data. Annor Frempong *et al.* [41] concluded that pattern recognition using a multivariate discriminant function is a linear method and hence may not perform well in situations where the response relationship cannot be completely approximated by linear combinations. They thus proposed the use of proper neural network techniques for pattern recognition.

Boothe and Arnold [42] also employed an electronic nose technique to analyze the volatile compounds emitted from poultry meat samples. Their sensor used MOS based sensors. Their study revealed that the electronic nose was able to detect changes in the volatile compounds associated with chicken meat based on the storage time and temperature. Their technique was also found to give reproducible results even after 6 months, indicating that the electronic nose was reliable. They had performed a Principal Component Analysis technique (PCA) on the data they obtained from their electronic nose. They reported that the PCA maps they obtained were able to differentiate (classify) the smell patterns obtained from different poultry meat samples (fresh and stored) and also between the samples stored at different temperatures.

Blixt and Borch [43] obtained the same reproducible results in their tests using MOS sensor array based electronic nose for determining the spoilage of vacuum-packaged beef. They followed a multivariate regression analysis approach (PLS) to develop mathematical models to predict the degree of spoilage in vacuum packaged beef. They used information regarding to the sensorial traits (like acidic, sulphurous and spoilage odors) and the sensor signals to obtain the weighted regression coefficients and the r^2^ values from the partial least squares regression (PLS) analysis. The best prediction model they developed with the data obtained had an r^2^ value of 0.94.

Di Natale *et al.* [44] described an electronic nose which employed a quartz microbalance (QMB) sensor coated with metallo-porphyrins and related compounds. Their electronic nose had eight QMB sensors each coated with different materials. For interpreting the resulting aroma patterns obtained, these researchers made use of a number of chemometrics based methods (principal component analysis and cluster analysis) and neural network based (feed forward back propagation trained networks, self organizing maps, adaptive resonance theory based networks) methods. They conducted their experiments at room temperature with an atmospheric relative humidity of about 40%. Their electronic nose system was found to be sensitive to organic compounds such as organic acids, alcohols, amines, sulphides and carbonyls which are some of the chief volatile components emitted during the breakdown of the chemical compounds in meat. Di Natale *et al.* [44] informed that their sensor was able to classify veal meat and codfish according to their storage days and can be correlated with the freshness of the products.

The spoilage of raw meat caused by microbiological processes taking place during storage represents a great problem in the meat industry and there is considerable waste in storage and handling of meat. Funazaki *et al.* [45] used one single semiconducting metal oxide gas sensor to determine freshness of sirloins from Holstein bulls. The sensor was specially designed to have a high specificity and sensitivity to ethyl acetate, one of the major components produced during the early stage of bacterial putrefaction in meat. The sensor had a linear range from l–200 ppm. They found a correlation factor of r^2^ = 0.8 between gas-sensor responses and bacterial counts. This example suggests that in some cases the sensor arrays of electronic noses have a too broad selectivity and that more specific and sensitive single gas sensors will be required for specific applications.

Arnold and Senter [46] analyzed the VOCs emitting from poultry by different bacteria species using an electronic nose and a GC-MS. They found the percent area under the curve for a selected number of predominant volatiles and reported them for each species of bacteria, which were inoculated in the poultry meat. The smell patterns, which were obtained from the 32 sensors in the electronic nose was reduced into two dimensions using multiple discriminant analysis technique. These patterns were then analyzed by cluster analysis (Sammon mapping) and then plotted as a map. An artificial neural network was used to classify the smell print data. Finally, the data obtained from the electronic nose and the GC-MS was used to compare the gases emitted from the different bacteria species. Their research showed promising results justifying the use of an electronic nose for classifying food products depending on their quality.

Grigioni *et al.* [47] have explained how an electronic nose system could be used to identify warmed over flavor (WOF) aroma in vacuum cook-in-bag/tray processed beef. They used multivariate analysis approach to classify their data into two groups depending on the WOF odor. They used a principal component analysis approach for data reduction. The trained artificial neural network model obtained by these researchers was able to classify the beef samples were significant WOF odor could be detected into class II with above 75% recognition confidence.

Neely *et al.* [48] used an electronic nose to distinguish between different types of meat. Their nose was equipped with semi-conducting polymer film (14 in number) sensors. The data from the electronic nose was analyzed by linear discriminant analysis method. The underlying technique employed by this method for discrimination is to form linear functions of the data to maximize the ratio of the between-group sum of squares to the within-group sum of squares. These linear functions are orthogonal. Once the linear functions are computed, the classification process is affected by finding out the Euclidean distance of an observation from the group of centroids, projected onto the subspace defined by a subset of the linear functions [49]. The observation is then assigned to the closest group. To evaluate the performance of this method, the “leave-one-out” cross validation method of estimating the centroids was used. Their tests showed that their electronic nose classified the meat types accurately.

Panigrahi *et al.* [49] have described in detail the data analysis technique used by them to analyze their output obtained from a metal oxide based electronic nose to classify beef strip loins stored at two different temperatures. They employed a fuzzy-c means clustering technique for their analysis. The area under the curve for each sensor response was used as the input dataset and two clusters were achieved. They achieved a maximum total classification accuracy of about 95% in classifying the meat samples into the two selected groups.

As known, the gas sensor array technology may as well be interesting to use in the monitoring of food processing. By adding starter cultures the desired aroma and flavor characteristics of fermented sausages are obtained. Eklov *et al.* [33] evaluated the use of a gas sensor array for monitoring the fermentation of sausages over a 52 hours period. They used a sensor array consisting of 10 MOSFET and four Taguchi (MOS) gas-sensors. With an artificial neural network model they could predict the fermentation process of 52 hour duration with an error of 2.7 hour. Berdague and Talou [25] used a chamber with one semiconductor gas sensor to monitor the maturation of unspiced sausages over a 90 day period. The sensor responses correlated with increased odor intensity with time and odor maximum at the late stage of maturation. They also analysed back fat from female and male pigs and could demonstrate significant differences in sensor responses related to sex. Accordingly, this technique should represent a rapid way to identify meat from male and female pigs.

**Figure 9.**
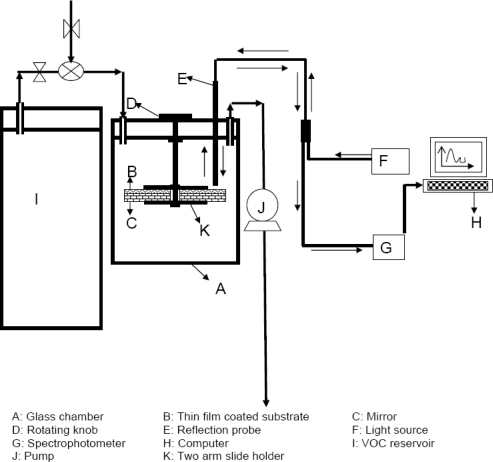
The schematic diagram of opto-electronic nose system for detection of VOC.

In a study done at North Dakota State University, Amamcharla and Panigrahi [50] conducted a research to select appropriate porphyrin and related compounds suitable for the characterization of beef spoilage, design and developing optical electronic nose system and characterization of one of the indicator compound, ethanol, associated with contamination of packaged beef. In their work, A prototype configuration of an optical sensing system in reflectance mode was designed and developed to evaluate the change in the thin film reflectance in the presence of ethanol vapor between 400 to 800 nm. It was found that CuP and RuOEPCO were sensitivity towards the 100 ppm ethanol vapor. A schematic representation of the set up used is shown in Figure 9.

Modeling quality changes of meat products during storage, e.g., sensory and/or microbiological spoilage by electronic nose systems have recently shown promising results in terms of monitoring quality changes. Hansen *et al.* [51] found that an electronic nose system comprising six MOS could detect the raw materials that led to unacceptable products (meat loaf), as determined by two types of sensory analyses. The electronic nose system could, however, not detect all the sensory unacceptable meat loaf samples due to changes in volatile composition after cooking. In a study on early stages of lipid oxidation, Olsen *et al.* [52] found that an electronic nose detected changes in pork back fat at the same time as a sensory panel.

Recently, a research done by Zhang *et al.* [53] was published. Referring to their work, separate sensors were used to construct a sensor matrix and the relationship between the matrix and beef quality was investigated. The test results show that some sensors could not be used for detecting beef quality and the relationship between the output of some sensors and the storage time of beef is linear, but the decay of the beef could not be detected clearly. Some sensors have no reaction to the fresh beef but have intense reaction to the decayed beef. A sensor matrix assembled from sensors that react differently to beef freshness could improve the reliability and the sensitivity of detection.

Continuous odor monitoring technologies are necessary to understand the complex odor-generating mechanisms within poultry housing as well as to identify strategies to reduce the impact of odor emissions on local communities. To evaluate electronic nose (EN) technologies for continuously assessing odor concentration in poultry housing, a mobile laboratory containing an electronic nose and an associated sample delivery system was deployed by Sohn *et al.* [54] to a commercial poultry farm and tested over a broiler production cycle. The results demonstrated that it was possible to develop a model to allow an electronic nose to provide a semi-continuous measurement of odor concentrations. The electronic nose was also able to demonstrate the influence of shed conditions on odor emissions.

A custom-built metal oxide-based olfactory sensing system was used by Balasubramanian *et al.* [55] to analyze the headspace from beef strip loins (M. *Longissimus lumborum*) stored at 4 °C and 10 °C. Classification models using radial basis function neural networks were developed using the extracted features and performance tested using leave-one-out cross validation method. The developed models classified the beef samples into two groups; “unspoiled” (<6.0 log_10_cfu/g) and “spoiled” (P 6.0 log_10_cfu/g) based on the microbial population. Maximum total classification accuracies above 90% were obtained for the samples stored at the two temperature values. Scaling the signals did have a positive influence in improving the classification accuracies obtained. Back propagation neural network prediction model using the pooled data (containing the area scaled feature) resulted in a R-squared of >0.70 between predicted and actual spoilage population from the 10 °C and 4 °C stored samples.

Modified atmosphere packaging is commonly applied to various fresh products including poultry meat to extend the shelf-life of the product. Inhibition of microbial growth is achieved by elevated carbon dioxide level and/or by minimised oxygen level in the package headspace. By Rajamaki *et al.* [56] the applicability of an electronic nose for the quality control of modified atmosphere (MA) packaged broiler chicken cuts was evaluated in different temperature regimes. The electronic nose results were compared with those obtained by microbiological, sensory and headspace GC analyses. The electronic nose could clearly distinguish broiler chicken packages with deteriorated quality from fresh packages either earlier than or at the same time as the sensory quality deteriorated. Concerning the microbiological quality, the counts of Enterobacteriaceae and hydrogen sulphide-producing bacteria were most consistent with the electronic nose results. The results indicated that the electronic nose was capable of detecting even early signals of spoilage in MA packed poultry meat.

Vestergaard *et al.* [57] conducted a study in Finland to investigate the predictability of an electronic nose system based on ion mobility regarding storage time as well as sensory quality changes during storage of a pork meat pizza topping product. The study included two independent test sets; “known” production samples and “unknown” samples purchased from a local supermarket (all samples stored at 51 °C after production or purchasing). Models for predicting storage time and sensory quality changes during storage from electronic nose data were estimated by projection of test set samples onto calibration models based on partial least square regression (PLSR). The results showed that storage time of “known” samples was very well predicted. Also, the storage time of “unknown” samples could be fairly well predicted. Sensory quality changes during storage were in general fairly well and significantly predicted for descriptors related to odor and color, whereas only few descriptors related to texture were found fairly well predicted. Descriptors found predictable for individual test sets clearly related to different stages of the storage time characterizing samples in the test sets, e.g., the test set of “known” samples comprised mainly of samples in the later stage of the storage time and thus descriptors mainly relating to the later stage of the storage time were well predicted, i.e., rancidity and greasy mouthfeel. Overall this study gave evidence of the electronic nose system to be a relevant device for future at or on-line implementation in quality control (QC) of a pork meat pizza topping product.

According to Barbri *et al.* [58] the reference method currently used for determining the spoilage status of meat is analysing the total count of bacteria and/or specific spoilage bacteria. An obvious drawback with such a bacteriological method is the incubation period of 1–2 days that is required for colony formation and, additionally, the lack of correlation between the degree of spoilage (from the sensorial point of view) and the total count of bacteria that is often observed [59]. Although, bacterial growth on meat samples has been extensively studied, methods based on the total count of bacteria that correlate well with shelf-life determination are still under investigation [60]. In spite of its drawbacks, bacteriological methods can be employed in many cases to define the desired product quality and are a good indicator of product safety. Furthermore, the results obtained from a bacteriological analysis can then be used to train alternative methods such as an electronic nose system. Barbri *et al.,* [58] developed an electronic nose for the quality control of red meat. Electronic nose and bacteriological measurements were performed to analyze samples of beef and sheep meat stored at 4 °C for up to 15 days. Principal component analysis (PCA) and support vector machine (SVM) based classification techniques are used to investigate the performance of the electronic nose system in the spoilage classification of red meats. The bacteriological method was selected as the reference method to consistently train the electronic nose system. The SVM models built classified meat samples based on the total microbial population into “unspoiled” (microbial counts <6 log_10_cfu/g) and “spoiled” (microbial counts 6 log_10_cfu/g). The preliminary results obtained by the bacteria total viable counts (TVC) show that the shelf-life of beef and sheep meats stored at 4 °C are 7 and 5 days, respectively. The electronic nose system coupled to SVM could discriminate between unspoiled/ spoiled beef or sheep meats with a success rate of 98.81 % or 96.43 %, respectively. To investigate whether the results of the electronic nose correlated well with the results of the bacteriological analysis, partial least squares (PLS) calibration models were built and validated. Good correlation coefficients between the electronic nose signals and bacteriological data were obtained, a clear indication that the electronic nose system can become a simple and rapid technique for the quality control of red meats.

The utility of chemosensor array (EN) signals of head-space volatiles of aerobically stored pork cutlets as a non-invasive technique for monitoring their microbiological load was studied by Horvath *et al.* [61] during storage at 4, 8 and 12 °C, respectively. The bacteriological quality of the meat samples was determined by standard total aerobic plate counts (TAPC) and colony count of selectively estimated *Pseudomonas* (PS) spp., the predominant aerobic spoilage bacteria. Statistical analysis of the electronic nose measurements were principal component analysis (PCA), and canonical discriminant analysis (CDA). Partial least squares (PLS) regression was used to model correlation between microbial loads and EN signal responses, the degree of bacteriological spoilage, independently of the temperature of the refrigerated storage. Sensor selection techniques were applied to reduce the dimensionality and more robust calibration models were computed by determining few individual sensors having the smallest cross correlations and highest correlations with the reference data. Correlations between the predicted and “real” values were given on cross-validated data from both data reduced models and for full calibrations using the 23 sensor elements. At the same time, sensorial quality of the raw cutlets was noted subjectively on faultiness of the odor and color, and drip formation of the samples. These preliminary studies indicated that the electronic nose technique has a potential to detect bacteriological spoilage earlier or at the same time as olfactory quality deterioration.

By Tikk *et al.* [62], a sensory analysis of meatballs was carried out to monitor the warmed-over flavor (WOF) development in cooked, cold-stored (at 4 °C for 0, 2 and 4 days) and reheated meatballs derived from *M. longissimus dorsi* (LD) and *M. semimembranosus* (SM) of pigs fed a standard diet supplemented with either 3% of rapeseed oil or 3% of palm oil. This was performed in combination with measurement of volatile compounds using a solid-state-based gas sensor array system (electronic nose) and gas chromatography/mass spectrometry together with measurement of thiobarbitoric acid reactive substances (TBARS). Subsequently, to elucidate the relations and predictability between the obtained data, the gas sensor responses were correlated with chemical (volatile and non-volatile secondary lipid oxidation products) and sensory data (flavor and odor attributes), using partial least squares regression modeling (PLSR). The TBARS, hexanal, pentanal, pentanol and nonanal all correlated to the sensory attributes associated to WOF formation. Moreover, the responses from eight of the MOS (metal oxide semiconductor) sensors within the electronic nose proved to be significantly related to WOF characteristics detected by both sensory and chemical analysis, while six of the MOSFET (metal oxide semiconductor field effect transistor) sensors were related to freshly cooked meat attributes determined by sensory analysis. The obtained results show the potential of the present gas sensor technology to monitor WOF formation in pork.

In a research conducted by Balasubramanian *et al.* [63], the changes in the headspace from stored beef strip loins inoculated with *Salmonella typhimurium* and stored at 20 °C were detected using an electronic nose system. Once the data was obtained six area-based features were extracted from the collected sensor data pertaining to the six metal oxide sensors present in the electronic nose. These extracted features were next dimensionally reduced by principal component analysis (PCA) and the independent components (IC) were extracted by FastICA package. The extracted independent components and principal components (PC) were compared by plotting them individually against the Salmonella population counts. A stepwise linear regression prediction model with the IC and PC as inputs was also built. The prediction model with IC as input performed better with an average prediction accuracy of 82.99%, and root mean squared error (RMSE) of 0.803. For the model using the PC as the input, the average prediction accuracy was 69.64% and the RMSE was 1.358. The results obtained suggest that the use of higher-order statistical techniques like ICA could help in extracting more useful information than PCA and could help in improving the performance of the sensor system. Further analysis needs to be carried out on larger datasets, and by using non-parametric data analysis techniques like artificial neural networks to build the prediction models from the ICA extracted components.

## 6. Conclusions

An “electronic nose” is a system originally created to mimic the function of an animal nose. However, this analytical instrument is more a “multi-sensor array technology” than a real “nose”. Whatever the sensor technology, it is still far from the sensitivity and selectivity of a mammalian nose. Therefore, its aim is not to totally replace either the human nose or other analytical methods. A sensory panel is necessary to define the desired product quality which can then be used to train the system. Traditional analytical methods such as GC-analysis will always be needed to determine qualitatively or/and quantitatively why one food sample differs from others. The “electronic nose” can only perform quick “yes or no” tests in comparison to other products. It could occasionally replace sensory analysis and even perform better than a sensory panel in routine work, or in cases where non-odorous or irritant gases need to be detected. Therefore, an “electronic nose” can be regarded as an interesting tool for a quick quality test in various food applications. However, before it can be treated as a completely reliable, industrial instrument, much improvement is still needed, such as a reduction of the dead volume, development of calibration methods, etc. Odor reception in biology and the design of electronic as well as bioelectronic noses are fascinating fields of current research and development. First attempts are promising to build electronic noses for monitoring concentrations of different molecules in mixtures or for characterizing odors. These systems are based on simple inorganic and organic sensor elements, utilize a modular sensor approach with arrays of different groups of sensor elements combined for one transducer principle, and make use of qualitative and quantitative pattern recognition evaluation schemes. The next steps in the development of bioelectronic noses are driven by (1) the need to monitor parameters which represent more directly impressions of human odor sensations and (2) the need for designing hybrid systems in order to make an optimum use of similarities and differences between technical and biological chemosensory systems.

At the end, this suggested that on-line sensors play a key role in the automation of meat control and processing. In near future when the basic issues of the gas-sensors have been solved, we will see more online gas-sensors implemented in the industry. For each application, however, technical problems have to be solved for implementation on-line. One interesting vision for the future would be to have a fully automated plattform of different kind of sensors to monitor the essential information required for the characterising of quality of the raw material, process or product. Gas-sensors would make up a vital part of such a multisensor system. This may be realised in the meat industry in the future.
